# Sonic Hedgehog Pathway Is Essential for Maintenance of Cancer Stem-Like Cells in Human Gastric Cancer

**DOI:** 10.1371/journal.pone.0017687

**Published:** 2011-03-04

**Authors:** Zhou Song, Wen Yue, Bo Wei, Ning Wang, Tao Li, Lidong Guan, Shuangshuang Shi, Quan Zeng, Xuetao Pei, Lin Chen

**Affiliations:** 1 Department of General Surgery, General Hospital of Chinese PLA, Beijing, China; 2 Stem Cell and Regenerative Medicine Lab, Beijing Institute of Transfusion Medicine, Beijing, China; Roswell Park Cancer Institute, United States of America

## Abstract

Abnormal activation of the Sonic hedgehog (SHH) pathway has been described in a wide variety of human cancers and in cancer stem cells (CSCs), however, the role of SHH pathway in gastric CSCs has not been reported. In this study, we investigated the possibility that abnormal activation of the SHH pathway maintained the characteristics of gastric CSCs. First, we identified cancer stem-like cells (CSLCs) from human gastric cancer cell lines (HGC-27, MGC-803 and MKN-45) using tumorsphere culture. Compared with adherent cells, the floating tumorsphere cells had more self-renewing capacity and chemoresistance. The cells expressing CSCs markers (CD44, CD24 and CD133) were also significantly more in tumorsphere cells than in adherent cells. More importantly, in vivo xenograft studies showed that tumors could be generated with 2×10^4^ tumorsphere cells, which was 100-fold less than those required for tumors seeding by adherent cells. Next, RT-PCR and Western blot showed that the expression levels of Ptch and Gli1 (SHH pathway target genes) were significantly higher in tumorsphere cells than in adherent cells. The results of quantitative real-time PCR were similar to those of RT-PCR and Western blot. Further analysis revealed that SHH pathway blocked by cyclopamine or 5E1 caused a higher reduction in self-renewing capacity of HGC-27 tumorsphere cells than that of adherent cells. We also found that SHH pathway blocking strongly enhanced the efficacy of chemotherapeutic drugs in HGC-27 tumorsphere cells in vitro and in vivo but had no significant effect in adherent cells. Finally, we isolated the tumorspheres from gastric cancer specimen, these cells also had chemoresistance and tumorigenic capacity, and SHH pathway maintained the gastric CSLCs characteristics of tumorsphere cells from primary tumor samples. In conclusion, our data suggested that SHH pathway was essential for maintenance of CSLCs in human gastric cancer.

## Introduction

CSCs play an important role in cancer development, this subpopulation within tumor possesses the characteristics of self-renewing capacity, chemoresistance and tumorigenic capacity thus may play a crucial role in the initiation, progression and recurrence of cancer. Examining and manipulating the biochemical pathways involved in those characteristics is one of the best ways of contributing to CSCs, leading to the development of novel drugs and treatment procedures. SHH pathway plays a critical role in many CSCs, such as glioblastoma stem cells [1], [2], CD34+ leukemic cells[3], [4] and breast CSCs[5], in addition, it is crucial to the development and homeostasis of gastric gland[6], abnormal activation of the SHH pathway could result in gastric cancer[7], [8], however, the role of SHH pathway in gastric CSCs is not clear.

SHH pathway in mammalian cells is mediated by ligands Shh[9]. In the absence of Shh, the transmembrane receptor patched (Ptch) inhibits the activity of another transmembrane protein, smoothened (Smo), resulting in inactivation of SHH pathway [9], [10]. Binding of Shh to Ptch abrogates the inhibitory effect of Ptch, and Smo is derepressed, thereby activating transcription factor Gli(Gli1,Gli2 and Gli3)[9], [10]. Gli1 is a strong positive activator of downstream target genes and is itself a transcriptional target of SHH pathway [11]. Therefore, Gli1 is considered a marker of SHH pathway abnormal activation [10], [12].

Recently, several types of CSCs or CSLCs have been identified and isolated from malignant tumors [13]–[25]. In gastric cancer, methodologically, it is difficult to isolate and amplify CSCs from cell lines or tumor tissue specimens. At the present time, CD44 appears to be the most useful marker for prospective purification of gastric CSCs, however, it is not highly specific for gastric CSCs [26].

In this study, we isolated CSLCs from human gastric cancer cell lines (HGC-27, MGC-803 and MKN-45) using tumorsphere culturing in serum-free medium and identified the self-renewal capacity, chemoresistance and tumorigenic capacity of the tumorsphere cells; next, we examined the expression of SHH pathway-related molecules, including Shh, Ptch, Smo, Gli1 and Gli2 in tumorsphere cells and adherent cells, especially the expression of Ptch and Gli1.Then, we blocked SHH pathway by cyclopamine or control tomatidine, 5E1 or control PBS to investigate whether the characteristics of CSLCs in tumorsphere cells were maintained by abnormal activation of the SHH pathway. Finally, we utilized primary gastric cancer samples to analyze the functional aspects of SHH pathway in gastric CSLCs.

Cyclopamine is a cell-permeable steroidal alkaloid. It disrupts cholesterol bio-synthesis and specifically antagonizes SHH signaling pathway through direct interaction with Smo (smoothened), a distant relative of G-protein-coupled receptors. It is a valuable tool for studying the involvement of SHH signaling in the development of various tumors. Tomatidine is a steroidal alkaloid that structurally resembles cyclopamine, but lacks the capacity to inhibit SHH signaling. It can be used as a negative control. 5E1 is an anti-Hh monoclonal antibody, which neutralizes the SHH and IHH activity.

## Results

### 1. Tumorsphere cells possessed the characteristics of CSLCs

**Figure 1:**
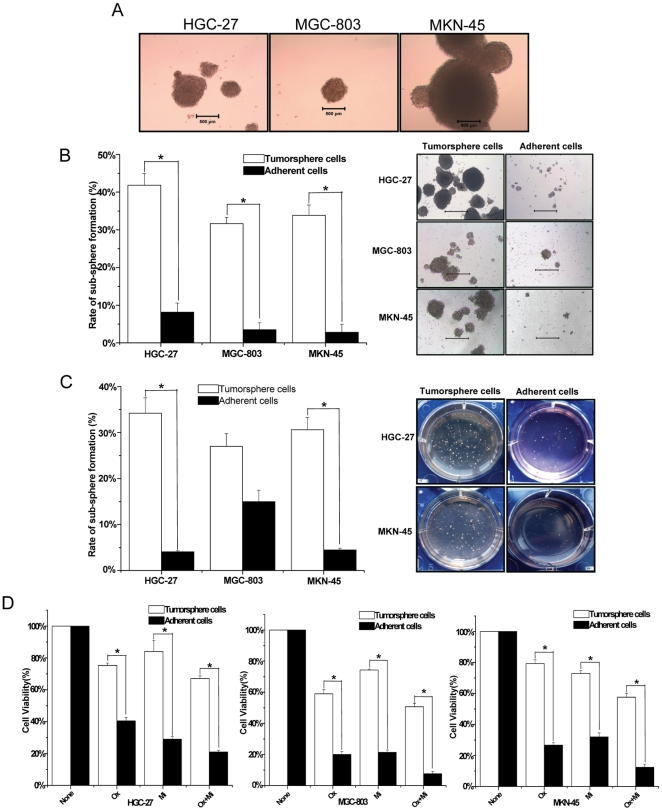
The CSCs characteristics of tumorsphere cells. (A) Tumorspheres formed after 14 days. (B) The sub-tumorspheres formation ability of tumorsphere cells (white bars) and adherent cells (black bars). * = P<0.05. Bars = 1000µm. (C) The colonies formation ability of tumorsphere cells (white bars) and adherent cells (black bars) under anchorage-independent conditions by soft agar assays. * = P<0.05. (D) Effect of 48 hours exposure of tumorsphere cells (white bars) and adherent cells (black bars) to 1000 µM chemotherapeutic drug. Ox = oxaliplatin, Mi = mitomysin. * = P<0.05.

We grew HGC-27, MGC-803 and MKN-45 adherent cells in serum-free medium described in the methods section. After 7 days, tumorspheres consisting of approximately 50 to 100 cells were observed (**Fig. 1A**).

The self-renewing capacity of these tumorspheres was assessed by dissociating them into single cell and growing at a clonal density of 1,000 cells per milliliter. After 2 weeks, single cell derived from HGC-27 tumorsphere cells generated sub-tumorspheres at 41.83%±3.13% compared with 8.17%±2.40% of adherent cells, moreover, in MGC-803 and MKN-45, the sub-tumorspheres formation rate of tumorsphere cells were also higher than that of adherent cells. (**Fig. 1B**). These data suggested that the self-renewing capacity of tumorsphere cells was significantly higher than that of adherent cells. Tumorsphere cells also showed an increased ability to form colonies under anchorage-independent conditions in a standard soft agar assay after 14 days in HGC-27 and MKN-45 (**Fig. 1C**).

We also performed a limiting dilution assay for spheroids formation using HGC-27 tumorsphere cells and adherent cells, respectively. As shown in **Table S1**, approximately 27–35% of tumorsphere cells could produce spheroids, while less than 5% of adherent cells could generate spheroids after 3 weeks culture. Therefore, in the HGC-27 tumorsphere cells, we could estimate that the spheroids-forming cells population comprised a maximum of 35%.

Chemoresistance is another characteristic of CSCs, so we investigated whether a difference in chemoresistance existed between tumorsphere cells and adherent cells. The results showed that, after 48 hours, tumorsphere cells demonstrated significantly greater resistance to drugs compared with adherent cells under the same conditions (**Fig. 1D**). We also assessed chemoresistance of HGC-27 tumorsphere cells and adherent cells to different concentrations of drugs, however, both tumorsphere cells and adherent cells did not show significantly chemoresistance to drugs in a dose-dependent fashion (**Fig. S1**).

**Figure 2:**
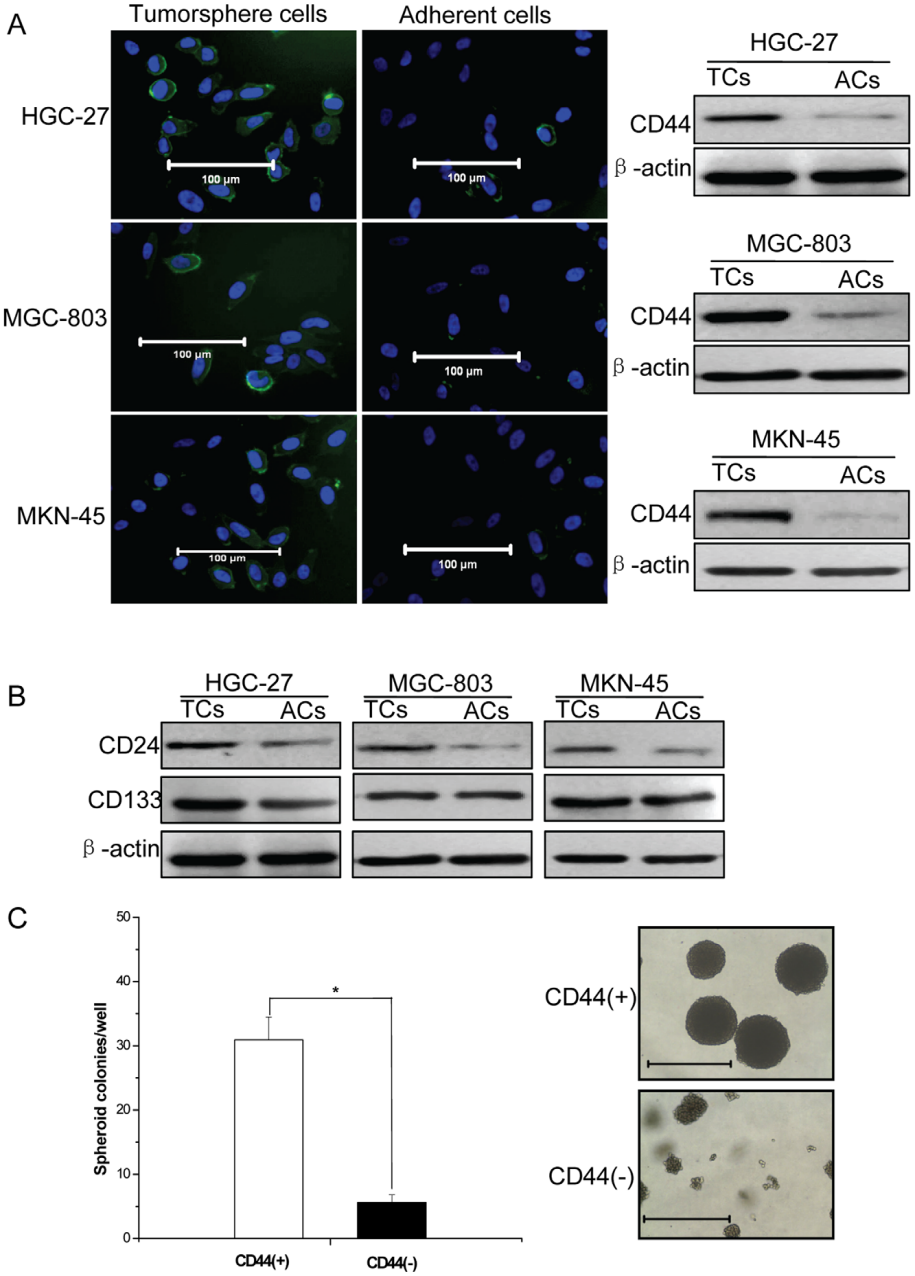
The expression of CSCs markers in tumorsphere cells and adherent cells. (A) Immunofluorescence (left panel) and western blotting (right panel) analysis of CD44 (green color) on mechanically dissociated tumorsphere cells and adherent cells. Nuclei were counterstained by DAPI (blue color). TCs = tumorsphere cells, ACs =  adherent cells. Bars = 100µm. (B) The expression of CD24 and CD133 in tumorsphere cells and adherent cells by western blotting, β-actin was provided as a control. (C) The tumorspheres formation capacity of HGC-27 CD44(+) cells and CD44(−) cells. * = P<0.05. Bars = 1000µm.

Next, we examined the expression of CD44 in dissociated tumorsphere cells and adherent cells using immunofluorescent assay and western blot. The results showed that the cells expressing CD44 were significantly more in tumorsphere cells than in adherent cells (**Fig. 2A**). Moreover, the expression of other CSCs markers(CD24 and CD133) in tumorsphere cells and adherent cells was examined by western blot, compared with tumorsphere cells, the expression of CD24 was significantly lower in adherent cells, while the expression of CD133 was similar (**Fig. 2B**). Further, we fractionated HGC-27 cell by FACS sorting for CD44, we found that CD44(+) cell fraction could generate more spheroid colonies compared with CD44(−) cell fraction, moreover, the size of spheroid colonies from CD44(+) cells was larger than those from CD44(−) cells(**Fig. 2C**).

### 2. Tumorsphere cells could generate tumors upon xenotransplantation

**Figure 3:**
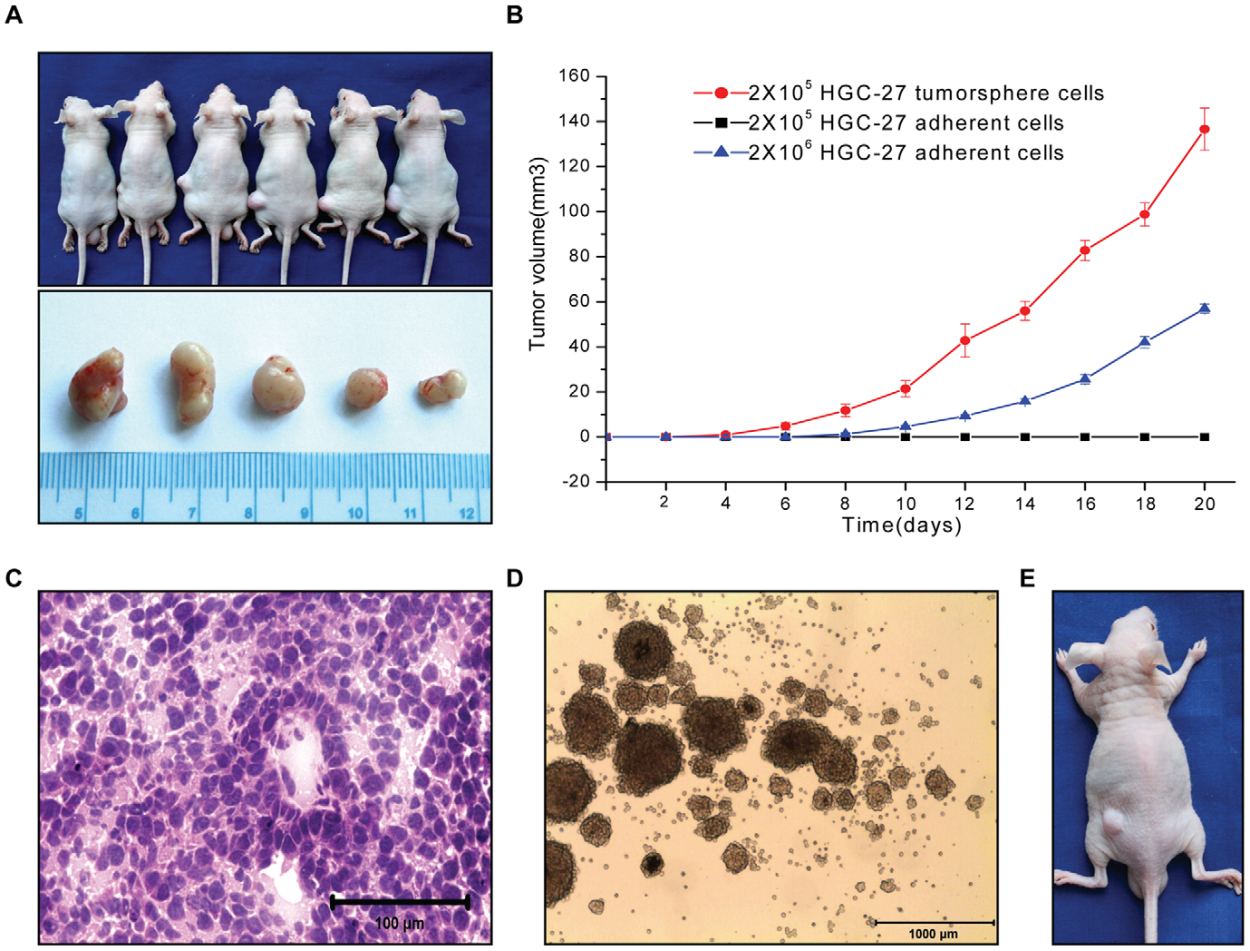
Subcutaneous tumor growth in nude mice. (A) Subcutaneous tumor growth in nude mice after injection of HGC-27 tumorsphere cells (2×10^5^) into the left rear flank of each mouse and equal numbers of adherent cells into the right rear flank of each mouse. (B) Size of subcutaneous tumor following injection of 2×10^5^ HGC-27 tumorsphere cells, 2×10^5^ HGC-27 adherent cells and 2×10^6^ HGC-27 adherent cells. (C) H&E staining analysis of xenografts derived from HGC-27 tumorsphere cells. Bar = 100 µm. (D) Tumorspheres derived from xenografts. Bar = 1000 µm. (E) Subcutaneous tumor growth in nude mice after injection of tumorsphere cells (2×10^5^) derived from xenografts.

For xenograft studies, equal number (2×10^4^, 2×10^5^, 2×10^6^, 2×10^7^) of freshly dissociated tumorsphere cells or control adherent cells was injected s.c. into each mouse (6 mice per group). The results showed that tumors were generated with 2×10^4^ tumorsphere cells, which was 100-fold less than those required for tumor seeding by adherent cells (**Table 1**). Moreover, tumorsphere cells generated subcutaneous tumors with larger volume and shorter time compared with those generated from adherent cells (**Fig. 3A,B**). Thus, subcutaneous injection of low number of tumorsphere cells confirmed that the spheroid cells retained stronger tumorigenic capacity in nude mice than adherent cells.

**Table 1:** Subcutaneous tumor growth in nude mice.

	HGC-27		MGC-803		MKN-45		Gastric tumor samples	
Cell injected	ACs	TCs	ACs	TCs	ACs	TCs	Bulk cells	TCs
2×10^4^	0/6	2/6	0/6	1/6	0/6	2/6	0/6	2/6
2×10^5^	0/6	5/6	0/6	4/6	0/6	5/6	0/6	5/6
2×10^6^	3/6	6/6	2/6	5/6	2/6	5/6	0/6	6/6
2×10^7^	5/6	6/6	3/6	6/6	4/6	6/6	2/6	2/6

Tumorsphere cells (TCs) have stronger tumorigenic capacity than adherent cells(ACs) and bulk cells.

H&E examination of xenografts derived from HGC-27 tumorsphere cells showed that these tumors closely resembled the original human tumor, mainly containing differentiated cells (**Fig. 3C**). Importantly, tumorspheres could be re-derived from the xenotransplanted tumors (**Fig. 3D**), and they could form tumor again (**Fig. 3E**). These data therefore indicated that the tumorsphere cells represented CSLCs that had tumorigenic capacity.

### 3. SHH pathway was higher expressed in tumorsphere cells than in adherent cells

**Figure 4:**
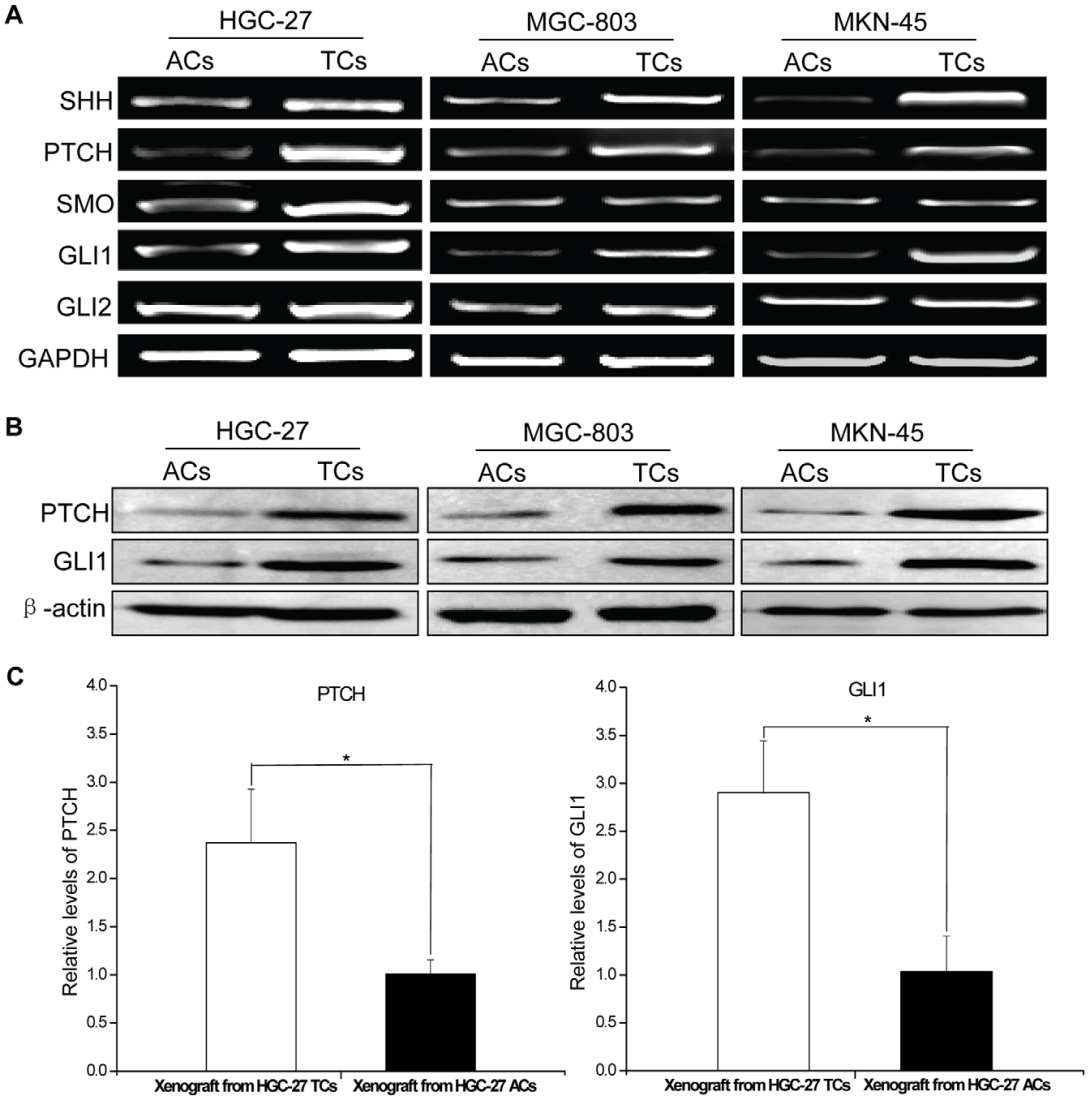
The expression of SHH pathway-related molecules. (A) The mRNA expression of Shh, Ptch, Smo, Gli1 and Gli2 in tumorsphere cells and adherent cells was analyzed by RT-PCR. The expression of GAPDH was used as a control. (B) The protein expression of SHH pathway target genes (Ptch and Gli1) was analyzed by Western blotting, β-actin was provided as a control. TCs = tumorsphere cells, ACs =  adherent cells. (C) Quantitative real-time RT–PCR analyses of SHH pathway target genes (Ptch and Gli1) showed the same results of the semi-quantitative RT–PCR. Results were calculated as meant+s.d. values from triplicate measurements of three separate experiments. TCs = Tumorsphere cells, ACs = adherent cells.

To investigate the link between SHH pathway and CSLCs characteristics of tumorsphere cells, we first examined the mRNAs expression of SHH pathway-related molecules, including Shh, Ptch, Smo, Gli1 and Gli2 by RT-PCR. The results showed that all of the mRNAs were high expressed in HGC-27 tumorsphere cells. Compared with tumorsphere cells, the expression levels of Shh, Ptch, Smo and Gli1 were significantly lower in adherent cells, while the expression levels of Gli2 were similar to tumorsphere cells. In MGC-803 and MKN-45, the expression levels of Shh, Ptch and Gli1 were also significantly lower in adherent cells than in tumorsphere cells, while the expression levels of Smo and Gli2 had no significant difference between adherent cells and tumorsphere cells (**Fig. 4A**).

In SHH pathway, Ptch and Gli1 are target genes, they are considered as important markers of SHH pathway abnormal activation. So, we further examined the expression levels of Ptch and Gli1 by Western blot and quantitative real-time PCR.

When using Western blot, we found that the expression levels of Ptch and Gli1 were also higher in tumorsphere cells than in adherent cells (**Fig. 4B**). We next assessed the expression levels of Ptch and Gli1 in xenografts from HGC-27 by quantitative real-time PCR. Quantitative real-time PCR analyses of SHH target genes (PTCH and GLI1) showed the same results of above semi-quantitative RT–PCR (**Fig. 4C**).

### 4. SHH pathway blocking reduced the self-renewing capacity and chemoresistance of HGC-27 tumorsphere cells

To test for a possible role of SHH pathway in self-renewing capacity of tumorsphere cells, we used cyclopamine or control tomatidine, 5E1 or control PBS to block the pathway.

**Figure 5:**
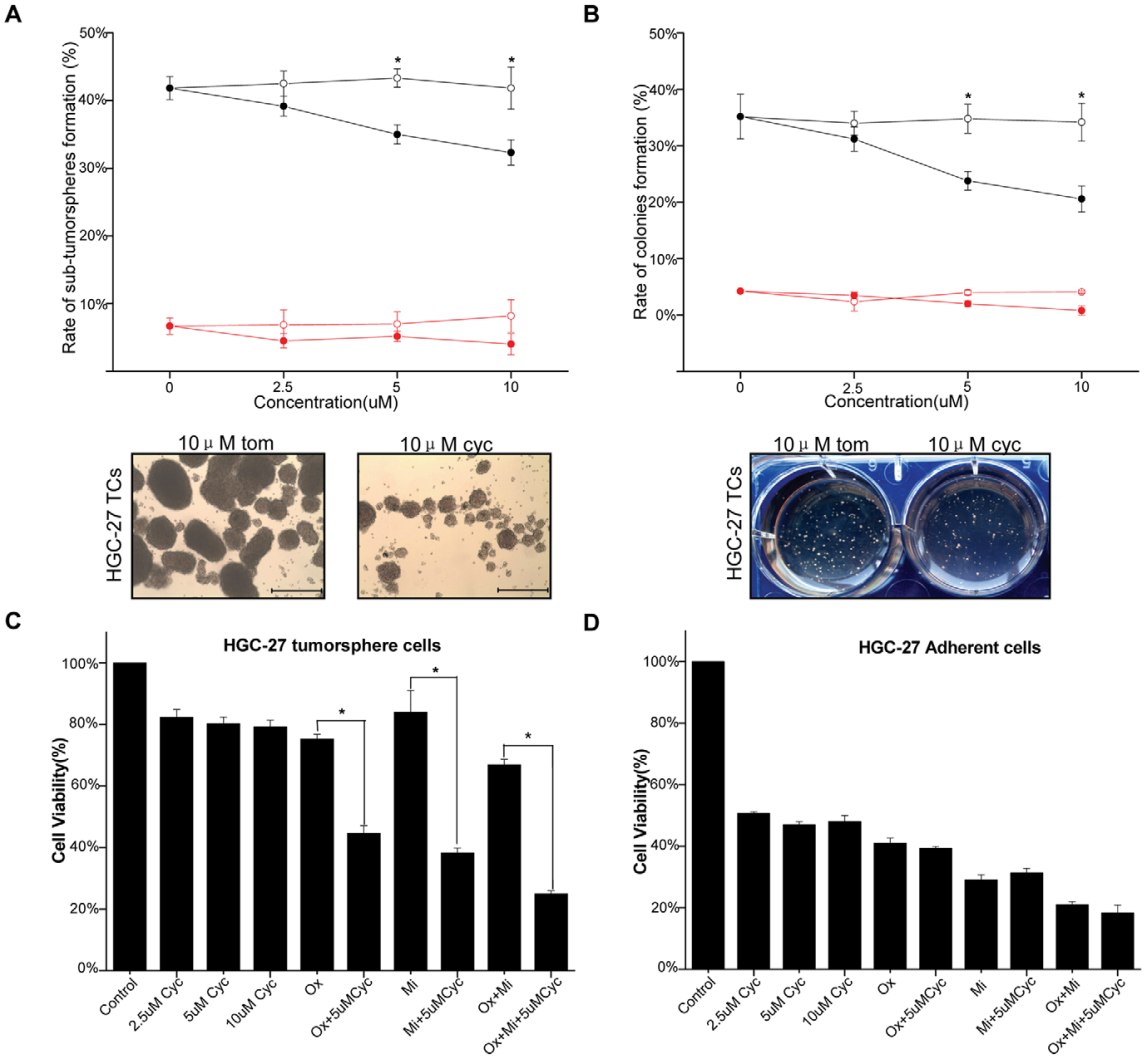
Effect of SHH pathway blocking on self-renewing capacity and chemoresistance. (A) The sub-tumorspheres formation ability of HGC-27 tumorsphere cells and adherent cells treated with different concentrations of cyclopamine or control tomatidine (open circle = cyclopamine;closed circle = control tomatidine). Black lines are tumorsphere cells, red are adherent cells. * = P<0.05. TCs = tumorsphere cells. Bars = 1000µm. (B) The colonies formation of HGC-27 tumorsphere cells and adherent cells treated with different concentrations of cyclopamine or control tomatidine under anchorage-independent conditions (open circle =  cyclopamine;closed circle = control tomatidine). Black lines are tumorsphere cells, red are adherent cells. * = P<0.05. TCs = tumorsphere cells. (C) Percentage of cell viability was analyzed on HGC-27 tumorsphere cells and adherent cells (D) after exposure to different concentrations of cyclopamine or 5 uM cyclopamine with different drugs for 48 hours. Control group is tomatidine. Cyc = cyclopamine, Ox = oxaliplatin, Mi = mitomysin. * = P<0.05.

The results showed that cyclopamine treatment led to a significantly reduction in capacity for the formation of sub-tumorspheres in HGC-27 tumorsphere cells in a dose-dependent fashion, but no such effect was observed in adherent cells (**Fig. 5A**). We further evaluated whether treatment with cyclopamine would affect the colonies formation capacity of HGC-27 tumorsphere cells by anchorage -independent growth assay. Similarly, the capacity to form colonies was reduced more in the tumorsphere cells in a dose-dependent fashion relative to the adherent cells (**Fig. 5B**).

Next, we investigated the effect of SHH pathway on chemoresistance of HGC-27 tumorsphere cells, we treated the dissociated cells with cyclopamine or control tomatidine for 48 hours, and then drug resistance of tumorsphere cells was evaluated. When cyclopamine was followed by drugs, the treatment resulted in a significantly enhanced overall cell death rate. Best results were obtained when cyclopamine was combined with Oxaliplatin plus Mitomycin (**Fig. 5C**). No such synergistic effect was observed in adherent cells after exposure to drugs with cyclopamine for 48 hours (**Fig. 5D**).

We also assayed the effect of different concentrations of cyclopamine (2.5 uM, 5 uM, 10 uM) on cell death. Results showed that cyclopamine alone to tumorsphere cells could make a small but consistent decrease in the viable cell number. Compared with HGC-27 tumorsphere cells, the effect of cyclopamine on cell death to adherent cells was more significance (**Fig. 5C,D**).

When SHH pathway was blocked by 5E1 or control PBS, the results of self-renewing capacity and chemoresistance of tumorsphere cells and adherent cells were similar to those blocked by cyclopamine or control tomatidine (**Fig. S2**).

### 5. SHH pathway blocking enhanced tumor response to drugs in vivo

**Figure 6:**
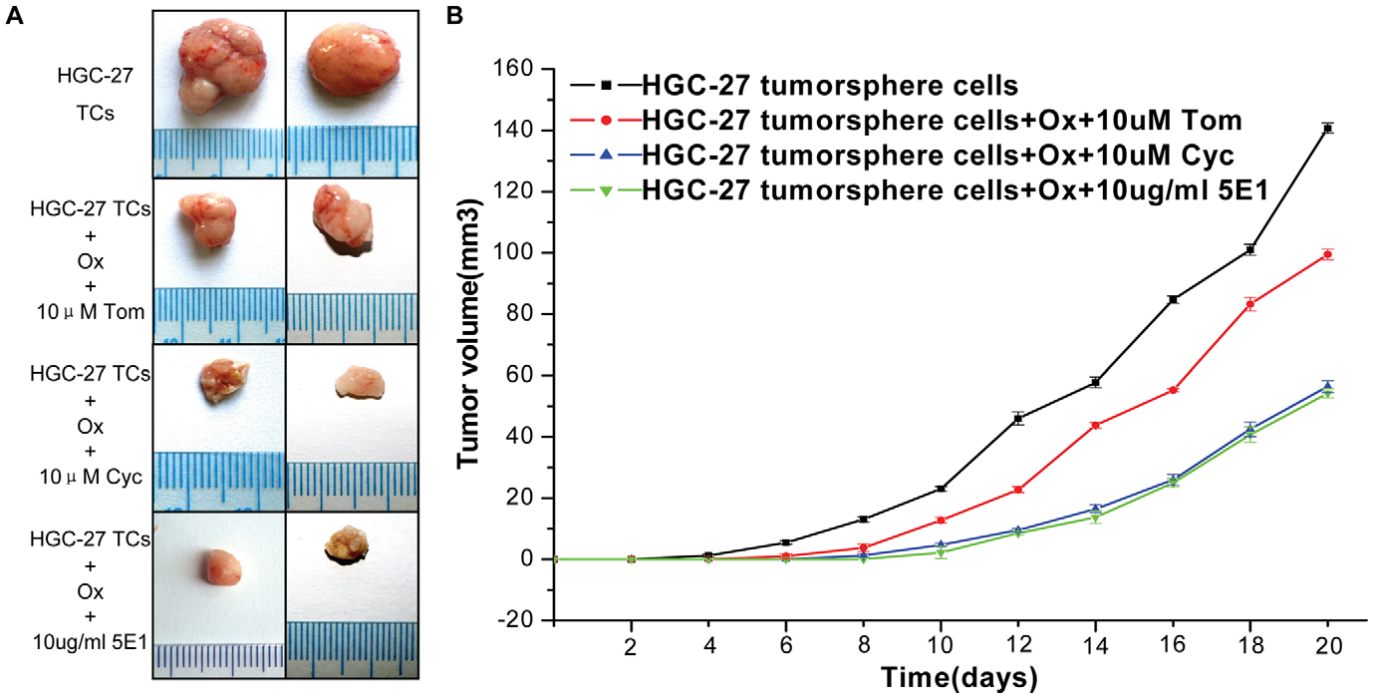
Effect of SHH pathway blocking on tumor response to drugs in vivo. (A) Subcutaneous tumors derived from 2×10^5^ HGC-27 tumorsphere cells grown in nude mice 4 weeks after i.p. treatment with PBS or oxaliplatin in combination with 10 µM control tomatidine at the left rear flank around the tumor, or oxaliplatin in combination with 10µM cyclopamine at the right rear flank around the tumor, or oxaliplatin in combination with 10 ug/ml 5E1 at the right rear flank. Tom = tomatidine, Cyc = cyclopamine, Ox = oxaliplatin. TCs = tumorsphere cells. (B) Tumor size derived as in (A) after 20 days. Tom = tomatidine, Cyc = cyclopamine, Ox = oxaliplatin.

To test this hypothesis in vivo, we analyzed whether SHH pathway blocking enhanced chemotherapy efficacy, cyclopamine or control tomatidine was used to inhibit SHH pathway responses. HGC-27 tumorsphere cells were allowed to grow into both rear flanks of nude mice until the larger diameter of tumor reached about 2 mm. Mice were then treated twice a week for 3 weeks with cyclopamine or control tomatidine 24 hours before Oxaliplatin delivery. In all treated mice, tumors treated with Oxaliplatin were significantly smaller than those no treated with Oxaliplatin. Moreover, tumors showed significantly greater tumor growth inhibition when they were treated with Oxaliplatin in combination with cyclopamine, the tumor response to chemotherapeutic drug was enhanced by cyclopamine (**Fig. 6A,B**). We also investigated whether SHH pathway blocked by 5E1 or control PBS enhanced chemotherapy efficacy in vivo, the results showed that 10 µg/ml 5E1 treatment led to a significantly greater tumor growth inhibition than those only treated with Oxaliplatin (**Fig. 6A,B**).

These data suggested that although standard chemotherapeutic drugs delayed tumor outgrowth during the treatment, when they were combined with cyclopamine or 5E1, the efficacy of chemotherapy was significantly enhanced and also more sustained after treatment interruption.

Next, to investigate whether blocking SHH pathway in vivo had a specific effect on HGC-27 tumorsphere cells. Mice were only treated cyclopamine at the right rear flank or control tomatidine at the left rear flank (twice a week for 3 weeks) when tumors from HGC-27 tumorsphere cells reached about 2 mm, after 3 weeks, we performed a limiting dilution assay for spheroids formation using tumor cells treated with cyclopamine and treated with tomatidine, respectively. As shown in **Table S2**, approximately 9–15% of cells from xenotransplanted tumor treated with tomatidine could produce spheroids, while less than 2.5% of cells from xenotransplanted tumor treated with cyclopamine could generate spheroids. Therefore, blocking SHH pathway in vivo could reduce the numbers of gastric CSLCs.

### 6. SHH pathway maintained the gastric CSLCs characteristics of tumorsphere cells from primary tumor samples

To determine whether SHH pathway could be involved in the gastric CSCs characteristics of tumorsphere cells from gastric cancer tissue, we utilized primary gastric cancer samples to analyze the functional aspects of SHH pathway in gastric CSLCs.

**Figure 7:**
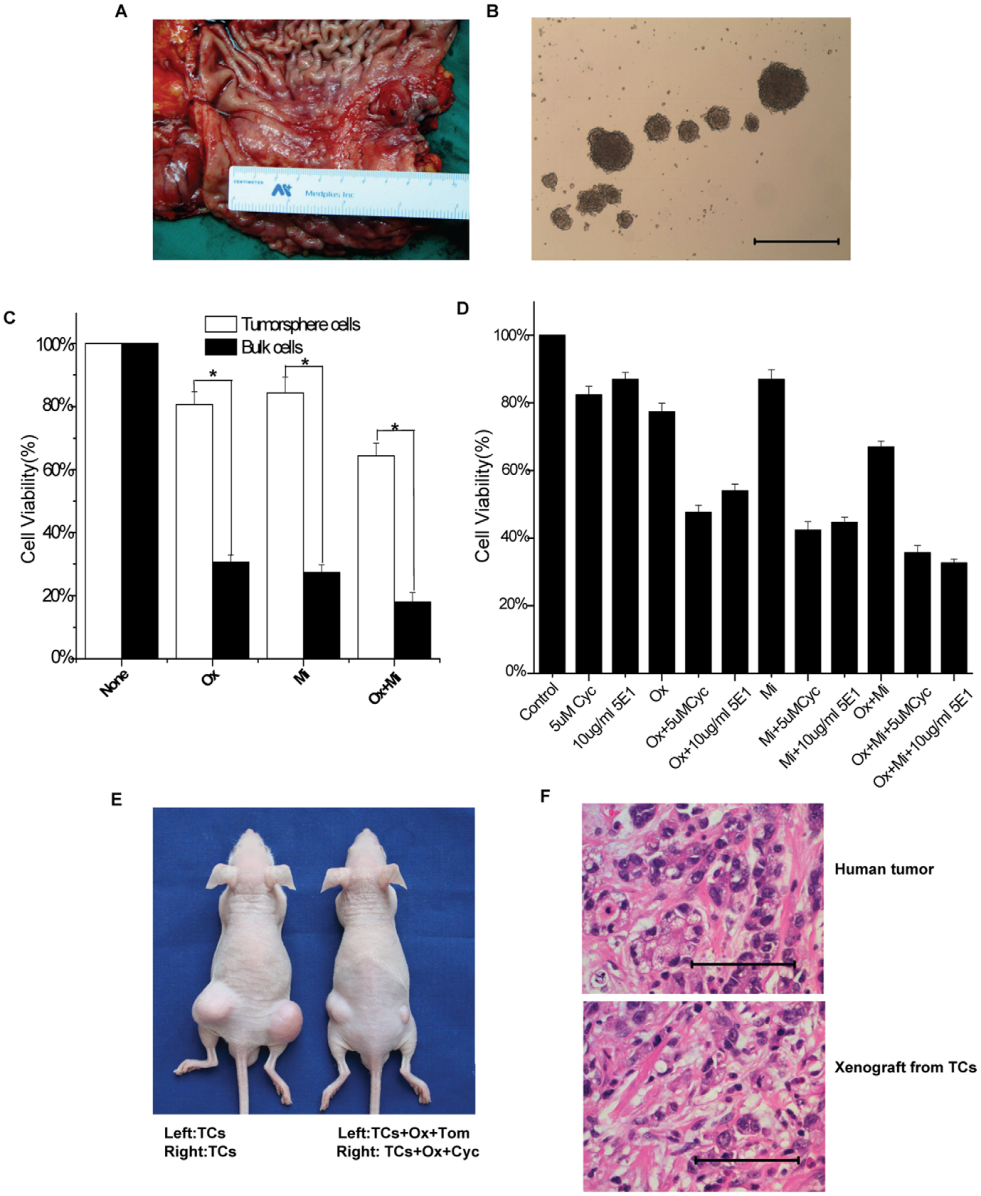
SHH pathway maintained the gastric CSLCs characteristics of tumorsphere cells from primary tumor samples. (A, B) Tumorspheres from gastric cancer specimen formed after 2 weeks later. Bars = 1000 µm. (C) Effect of 48 hours exposure of tumorsphere cells (white bars) and bulk cells (black bars) to 1000 µM chemotherapeutic drug. Ox = oxaliplatin, Mi = mitomysin. * = P<0.05. (D) Cell viability was analyzed on tumorsphere cells **from gastric cancer specimen** after exposure to cyclopamine or 5E1 with different drugs for 48 hours. Control group is PBS. Cyc = cyclopamine, Ox = oxaliplatin, Mi = mitomysin. (E) Subcutaneous tumors derived from 2×10^5^ tumorsphere cells **from gastric cancer specimen** after i.p. treatment with PBS or oxaliplatin in combination with 10µM control tomatidine at the left rear flank around the tumor, or oxaliplatin in combination with 10µM cyclopamine at the right rear flank around the tumor. Tom = tomatidine, Cyc = cyclopamine, Ox = oxaliplatin. TCs = tumorsphere cells. (F) H&E staining analysis of human tumor and xenografts derived from tumorsphere cells. Bar = 100 µm.

First, we grew freshly dissociated bulk cells from gastric cancer specimens in serum-free medium described in the methods section, after 10 days, tumorspheres were observed(**Fig. 7A,B**). Then we investigated chemoresistance and tumorigenic capacity of the tumorsphere cells from gastric cancer tissue. The results showed that tumorsphere cells from gastric cancer tissue demonstrated significantly greater resistance to drugs than bulk cells under the same conditions(**Fig. 7C**). Moreover, freshly dissociated bulk cells from gastric cancer specimens injected at 2×10^7^ cells per mouse were capable of establishing subcutaneous tumors, while as little as 2×10^4^ of the tumorsphere cells from gastric cancer specimens could form tumors (**Table 1**), the xenografts tumors that resulted from injection of tumorsphere cells presented the same histopathology features as their respective human tumor (**Fig. 7F**). These results showed that tumorsphere cells from gastric cancer tissue possessed the characteristics of CSLCs. Next, we observed the effect of SHH pathway blocking on chemoresistance of tumorsphere cells in vitro and tumor response to drugs in vivo. The results showed that cyclopamine or 5E1 could resulted in a significantly enhanced overall cell death rate (**Fig. 7D**) and the tumor response to drug (**Fig. 7E**).

## Discussion

CSCs share many characteristics with tissue stem cells, such as self-renewal, proliferation, chemoresistance, and are primarily responsible for sustaining the growth of tumors [27], [28]. Many investigators have used fluorescence-activated cell sorting to identify and isolate CSCs and determined that these cells can form spheres in specialized serum-free medium. In this study, we took the opposite approach by developing tumorsphere cells and then determining whether these cells acquired CSCs characteristics.

We used a defined serum-free medium consisting of EGF, FGF-2, B-27 supplement and N-2 supplement to maintain gastric cancer cell lines. These conditions had been used before to maintain endogenous neural stem/progenitors and tumor stem cells from human tumors and tumor cell lines. For example, Kondo et al. [24] characterized the C6 rat tumor cell line in vitro and in vivo and observed a small population of stem-like cells derived from the well-characterized side population. Setoguchi et al. [29] demonstrated cancer stem-like cell potential in other cancer cell lines, including the MCF7 breast cancer line, B104 neuroblastoma, and HeLa adenocarcinoma.

At our experiment, we used three gastric cancer cell lines (HGC-27, MKN-45 and MGC-803) to culture tumorspheres. HGC-27 cell line was established by culture of the metastatic lymph node from a gastric cancer patient diagnosed histological as undifferentiated carcinoma. MKN-45 was established from the poorly differentiated adenocarcinoma of the stomach of a 62-year-old woman. MGC-803 is a epithelial poorly differentiated human gastric muco-adenocarcinoma cell line from a 74-year-old man.

Two different approaches have typically been used to identify CSCs in published studies [30], [31]. One is an in vitro method termed “spheroid colony formation,” and another is in vivo method involving implantation of candidate CSCs under the skin of immunodeficient mice.

The former method involves culturing candidate CSCs in culture dishes specially coated for noncell attachment with serum-free media containing epidermal growth factor and basic fibroblast growth factor. The growth of spherical colonies after a few weeks is considered indicative of self-renewal ability, and would be consistent with a CSC phenotype. The latter method—growth of cells in immunodeficient mice—is needed to demonstrate true tumorigenicity and is generally regarded as the gold standard for proving existence of CSCs. A number of studies have suggested that these two approaches generally provide similar results in evaluating candidate CSCs for many solid tumors.

At present, gastric CSCs have not specific surface makers, so, we developed tumorsphere cells and then determined whether these cells acquired CSCs characteristics, including self-renewing capacity, chemoresistance and tumorigenic capacity.

Our data showed that gastric cancer cell lines (HGC-27, MGC-803, MKN-45) grown in a defined serum-free medium could form tumorspheres that contained cells behaving like CSLCs. In vitro, tumorsphere cells showed an increased ability to form colonies in soft agar under anchorage-independent conditions and an increased ability to form sub-tumorspheres. We also showed that the self-renewal ability to form sub-tumorspheres of HGC-27 tumorsphere cells had a increase at passages 2 to 8 (from 44.5% up to 70.5%), and then it maintained a relatively stable proportion after 8 or more passages when grown at clonal density (**Fig. S3**). These data suggested that the proliferative potential of tumorsphere cells was maintained after extended passages. In addition, tumorsphere cells in vitro were more resistant to Oxaliplatin and Mitomycin compared with adherent cells, these data suggested that tumorsphere cells consist of enriched numbers of CSLCs, which potentially made them less susceptible to the actions of both Oxaliplatin and Mitomycin. In vivo, tumorsphere cells could generate tumors upon xenotransplantation at a higher rate than adherent cells. Moreover, tumorsphere cells generated subcutaneous tumors with larger volume and shorter time compared with those generated from adherent cells, Importantly, tumorspheres could be re-derived from the xenotransplanted tumors, and they could form tumor again, these data suggested that tumorspheres were enriched in CSLCs.

In our study, adherent cells presumably contained a relatively low percentage of CSCs, as no method for isolation or removal of these cells was used. It may be argued that it is this small population of CSCs that is responsible for tumor formation. Regardless, more CSCs were evident in the tumorsphere cells, which may explain its more aggressive behavior.

Next, we investigated whether the characteristics of CSLCs in tumorspheres were maintained by abnormal activation of the SHH pathway. First, we demonstrated that mRNA expression levels of SHH pathway-related molecules (Shh, Ptch and Gli1) were significantly higher in tumorsphere cells than in adherent cells, while Gli2 was high expressed in both cells. We further examined the expression levels of SHH pathway target genes (Ptch and Gli1) by Western blot and quantitative real-time PCR, the results were similar to those of RT-PCR. Second, inhibition of SHH pathway activity by cyclopamine or 5E1 led to decreased self-renewal capacity and increased sensitivity to drugs in HGC-27 tumorsphere cells, but these effects were not pronounced in adherent cells. In vivo, in all treated mice, tumors treated with Oxaliplatin were significantly smaller than those no treated with Oxaliplatin, after treatment with cyclopamine or 5E1, the tumor response to chemotherapeutic drugs was enhanced. These data suggested that although standard chemotherapeutic drugs delayed tumor outgrowth during the treatment, when they were combined with cyclopamine, the efficacy of chemotherapy was significantly enhanced and also more sustained after treatment interruption.

Finally, we utilized primary gastric cancer samples to analyze the functional aspects of SHH pathway in gastric CSLCs. Results showed that tumorsphere cells from gastric cancer specimen also had chemoresistance and tumorigenic capacity, moreover SHH pathway blocking reduced the chemoresistance of tumorsphere cells and enhanced tumor response to drugs in vivo.

As for chemoresistance, several features of CSCs may make them hard to eliminate. CSCs are relatively quiescent and this allows them to escape from chemotherapeutic regimens that typically target actively cycling cells. Moreover, as shown for their normal counterpart, CSCs have been proposed to exhibit high level expression of multidrug transporter family genes, likely resulting in more efficient efflux of chemotherapeutic drugs and innate multidrug resistance, In addition, signaling pathways, such as Wnt, Hedgehog or Notch, are dysregulated in CSCs leading to tumor development. The development of an efficient therapeutic approach would therefore require the identification of distinctive molecular pathways active in CSCs and the identification of agents that can either block CSC proliferation or induce CSC differentiation, thus enhancing sensitivity to chemotherapeutic drugs. So, important factors affecting the chemoresistance of CSCs are intrinsic factors. The microenvironment, or niche, may influence the ability of CSCs to proliferate, migrate or invade. The niche is an anchoring site for CSCs, and adhesion molecules or microenvironmental soluble molecules, including growth factors and cytokines, can significantly contribute to the refractoriness to therapy. Although inhibition of SHH pathway by cyclopamine or 5E1 would be a useful strategy, in our study, we demonstrated that receptor component Ptch and transcription factor Gli1 were also high expressed in tumorsphere cells. So, Ptch or Gli1 might be an alternative target for treatment of gastric cancer. One possible strategy is to use small interfering RNA (siRNA) or shRNA specific for Ptch or Gli1, but gastric CSLCs in tumorsphere cells have not been completely purified, we need to further purify the gastric CSLCs, so this work will be done in our further study.

In conclusion, we showed that SHH pathway could be involved in the self-renewal, proliferation, drug resistance and tumorigenic of tumorsphere cells and suggest that strategies aimed at inhibiting SHH pathways represent a rationale therapeutic approach to target gastric CSCs.

## Materials and Methods

### Animal Ethics Statement

Male athymic nude mice(nu/nu), 6 to 8 weeks old, were obtained from Beijing Vital-River Lab Animal Technology Co. Ltd (SCXK JING 2007-0001) and were housed under pathogen-free conditions in the barrier animal facility. All mice were conducted under approved guidelines of Laboratory Animal Center of Academy of Military Medical Sciences (SYXK JUN 2002-001).

### Culture of adherent cells, tumorspheres and sub-tumorspheres

Human gastric cancer cell lines (HGC-27,MGC-803 and MKN-45) obtained from Peking Union Medical College was cultured in 1640 medium containing 10% fetal bovine serum (FBS) and plated at the density of 1×10^6^ live cells per 75-cm^2^ flask. When the cells attached, we passaged them upon confluence. Tumorspheres were derived by placing the adherent cells into serum-free 1640 culture medium containing 1% N-2 supplement (Invitrogen), 2% B-27 supplement (Invitrogen), 1% antibiotic mixture (Gibco), 20 ng/ml human FGF-2(Chemicon), and 100 ng/ml EGF (Chemicon), and the adherent cells were plated in 24-well ultra-low attachment plate (Corning) at 500 cells per well. 2 weeks later, plates were analyzed for tumorspheres formation and were quantified using an inverted microscope (Olympus) at 40× and 100× magnification.

After primary tumorspheres reached the size of approximately 200–500 cells per sphere, the tumorspheres were dissociated at the density of 1,000 cells per milliliter and 100 µL single cell suspension was seeded in each well of 24-well ultra-low attachment plate (Corning) in serum-free medium described above. Each well was examined for single cell, and only the wells that contained single cell were marked. 2 weeks later, wells were analyzed for sub-tumorspheres formation. Normal adherent cells were used as control for the capacity to form sub-tumorspheres. To test the link between SHH pathway and the formation of sub-tumorspheres, we treated the dissociated cells with cyclopamine (2.5 µM, 5 µM, 10 µM) or control tomatidine, 5E1(10 ug/ml) or control PBS and observed the formation of sub-tumorspheres.

### Gastric cancer tissue specimen

The human fresh, sterile gastric cancer tissue specimens were obtained in accordance with the ethical standards of the institutional committee on human experimentation from 15 patients (age range 70–85 years) undergoing a gastric cancer resection, after obtaining informed consent from the patients (**Table S3**).

Then the specimens were immersed in 95% ethanol for 2–3 sec to avoid contamination, and washed with phosphate-buffered saline (PBS) and antibiotics several times to remove the blood. After these, the surface of the cancer tissue specimens was removed and the inner parts were cut into 1–3 mm^3^-sized pieces. Then they were digested in collagenase and dispase solution at 37°C, after 4 h incubation, the solution containing the digested tissues was centrifuged to remove the collagenase solution. The cell pellet was washed once with 1640 medium before being suspended into serum-free 1640 medium containing 1% N-2 supplement, 2% B-27 supplement, 1% antibiotic mixture, 20 ng/ml human FGF-2, 100 ng/ml EGF and plated in 24-well ultra-low attachment plate. All cultures were incubated at 37°C in incubators supplied with humidified air and 5% CO_2_.

### Anchorage-independent growth assay

For observing the self-renewing capacity further, soft agar assay was used to determine the colonies formation of tumorsphere cells and adherent cells under anchorage-independent conditions. Each well of a six-well plate was coated with 1 mL of 10% FBS 1640 medium with 1% agarose. After 20 min of incubation at 37°C, equal numbers (500) of tumorsphere cells or adherent cells were added in 1 mL of 10% FBS 1640 medium with 0.5% agarose. To test the link between SHH pathway and the colonies formation under anchorage-independent conditions, we treated the dissociated cells with cyclopamine (2.5 µM, 5µM, 10µM) or control tomatidine, 5E1(10 ug/ml) or control PBS. Cells were incubated for 14 days under standard conditions (37(C, 5% CO2) and with the addition of 300µL of medium every 3 days to hydrate the exposed agarose. At the end of the incubation period, wells were examined under a light microscope and the number of colonies larger than 50µm was counted per well.

### Immunofluorescent analysis

For immunofluorescent staining, mechanically dissociated tumorsphere cells or adherent cells were fixed in 4% paraformaldehyde (Sigma) for 15–20 min at room temperature. Cells were washed three times with PBS and incubated for 10 min in blocking buffer which contained 10% goat serum, then incubated with FITC-conjugated anti-human CD44 (Biolegend) diluted at 1∶50 overnight at 4(C. Nuclei were visualized by DAPI (Sigma). PBS was used to replace the primary antibody and served as a negative control.

### Chemoresistance assay

Rates of resistance to drugs were assessed using MTT assay. Briefly, 2000 healthy tumorsphere cells or adherent cells per well were plated in 96-well plates in 200µL 1640 medium (4 wells per group) with 1000µM chemotherapeutic drugs (Oxaliplatin, Mitomycin or Oxaliplatin plus Mitomycin) or control PBS. At each time point (0, 24, 48 h), 20µL MTT solution was added to each well and the plate was incubated for 4 hours at 37°C, then the medium was replaced by 150 µL DMSO. To assess the effect of SHH pathway on drug resistance of tumorsphere cells or adherent cells, we treated the dissociated cells with cyclopamine (5 µM) or control tomatidine, 5E1 (10 ug/ml) or control PBS for 48 hours. MTT assay is based on mitochondrial conversion of MTT to yellowish formazan, being indicative of the number of viable cells. The number of viable cells was evaluated by absorbance OD450 nm (Abs) using Model 680 microplate reader.

### Western blot analysis

For Western blot analyses, protein was harvested from cells plated to 70% to 80% confluence. Tumorsphere cells or adherent cells were lysed directly in nuclear lysis buffer to collect whole cell extracts. Protein samples for Western blot were prepared by boiling after the addition of denaturing sample buffer. Then, proteins were separated using SDS-PAGE on an 8% or 15% gel, transferred onto nitrocellulose membrane by PowerPac (BIO-RAD). Membranes were incubated at 4°C overnight with primary antibody, and subsequently incubated with horseradish peroxidase –conjugated secondary antibodies for 1 h at room temperature. Finally, protein bands were visualized using chemiluminesce (Santa Cruz) exposure on BioMax film (Kodak). The following concentrations were used for primary antibodies: anti-CD44 1∶100 (Santa Cruz), anti-CD24 1∶100 (Santa Cruz), anti-133 1∶100(Santa Cruz), anti-Ptch 1∶300 (Santa Cruz), anti-Gli1 1∶300 (Santa Cruz), anti-actin: 1∶1000 (Santa Cruz).

### RNA extraction, RT–PCR, and quantitative real-time PCR

Total RNA was extracted by the guanidine isothiocyanate–phenol extraction method using the Trizol reagent (Invitrogen) as specified by the manufacturer. Two micrograms of total RNA were reverse transcribed using AMV reverse transcriptase (TaKaRaBio) to produce cDNAs. Amplification was performed in a GeneAmp 2700 thermal cycler (Applied Biosystems). The semi-quantitative reverse transcription (RT)–PCR and quantitative real-time PCR were carried out using primer sequences listed in **Table 2**. For semi-quantitative RT–PCR, we showed data within linear range by performing 25–35 cycles of PCR. For quantitative real-time PCR, the expression level of each mRNA was normalized with that of GAPDH mRNA.

**Table 2:** Primers and conditions used for RT-PCR and real-time PCR.

Gene	RT-PCR	Real-time PCR
SHH	5′-CGCACGGGGACAGCTCGGAAGT-3′ 5′-CTGCGCGGCCCTCGTAGTGC -3′	
PTCH	5′-GCCGTGCCCGTGGTCAT-3′ 5′-CCCATTGAGAACGCCGAGGAT-3′	5′-TCTCCAATCTTCTGGCGAGT-3′ 5′-TGGGATTAAAAGCAGCGAAC-3′
SMO	5′-GAAGTGCCCTTGGTTCGGACA-3′ 5′-CCGCCAGTCAGCCACGAAT-3′	
GLI1	5′-CAGGGAGTGCAGCCAATACAG-3′ 5′-GAGCGGCGGCTGACAGTATA-3′	5′-TCTCAAAGTGGGAGGCACAA-3′ 5′-CCCTTAGGAAATGCGATCTG-3′
GLI2	5′-AGAAGCAGCGCAATGACGTG-3′ 5′-GTCATCCAGTGCCGTCAGGT-3′	

### S.c. xenograft model

For xenograft studies, equal number (2×10^4^, 2×10^5^, 2×10^6^, 2×10^7^) of freshly dissociated cells was suspended in 200 µL PBS, the tumorsphere cells were injected s.c. into the left rear flank of each mouse (6 mice per group) and the adherent cells were injected s.c. into the right rear flank of each mouse, we examined the tumorigenic capacity of tumorsphere cells and adherent cells. Next, tumorsphere cells were allowed to grow into the both rear flanks of nude mice until the larger diameter of tumor reached about 2 mm, mice were then i.p. treated with PBS or oxaliplatin (0.25 mg/kg, once a week for 4 weeks) in combination with cyclopamine (10 µM, twice a week for 3 weeks) at the right rear flank and control tomatidine (10 µM, twice a week for 3 weeks) at the left rear flank, or in combination 5E1(10 ug/ml, twice a week for 3 weeks) at the right rear flank and in combination with control PBS(10 ug/ml, twice a week for 3 weeks) at the left rear flank, cyclopamine and tomatidine, or 5E1 and PBS were injected s.c. around the tumor. When tumors in the control group exceeded 1.5 cm in the larger diameter, mice were killed by CO^2^ asphyxiation according to protocol, and the tumors were excised. Tumors were weighed and measured, and a portion of each was placed in 10% formalin for immunohistochemistry and H&E staining. Tumor size was calculated every 2 days using the formula: (π/6)×larger diameter×(smaller diameter)^2^.

### H&E

The excised tumors from male athymic nude mice were fixed in 10% formalin, embedded in paraffin, and processed by standard histological methods. From each selected paraffin block, 5µm serial sections were cut. Then tissue sections were stained for H&E to assess morphology.

### Statistical analysis

All experiments were repeated at least three times and representative results are presented. Where applicable, quantitative data were presented as means (SD. Tumorsphere formation and tumor growth curves were analyzed by ANOVA with an SPSS10.0 statistical software. Two groups were compared by the Student's *t*-test, P<0.05 was considered significant.

## Supporting Information

Figure S1 **The chemoresistance of HGC-27 tumorsphere cells and adherent cells to different concentrations of drugs.** Figure showed that HGC-27 tumorsphere cells demonstrated significantly greater resistance to different concentrations (125µM, 250µM, 500µM and 1000µM) of drugs (Oxaliplatin, Mitomycin) compared with the adherent cells after 48 hours, however, both tumorsphere cells and adherent cells did not show significantly chemoresistance to drugs in a dose-dependent fashion. White bars = 1 day. Black bars = 2 day.
(TIF)

Figure S2 **Effect of anti-SHH antibody 5E1 on the self-renewing capacity and chemoresistance.** Figure showed that 10µg/ml 5E1 treatment did not led to decrease in the capacity for the formation of sub-tumorspheres in HGC-27 adherent cells but a significantly greater reduction (13.5%) in HGC-27 tumorsphere cells. Similarly, the capacity to form colonies in soft agar was reduced more in the HGC-27 tumorsphere cells (12.6%) relative to the adherent cells. The effect of 5E1 on cell death to HGC-27 adherent cells was more significance than tumorsphere cells, but no such synergistic effect was observed in adherent cells after exposure to drugs with 5E1 for 48 hours. When 5E1 was followed by drugs in HGC-27 tumorsphere cells, the treatment resulted in a significantly enhance overall cell death rate. Best results were obtained when 5E1 was combined with Oxaliplatin plus Mitomycin. Control group is PBS. * = P<0.05. Bars = 1000µm.
(TIF)

Figure S3 **The self-renewal ability to form sub-tumorspheres of HGC-27 tumorsphere cells during serial passages.** Figure showed that the self-renewal ability to form sub-tumorspheres of HGC-27 tumorsphere cells had a increase at passages 2 to 8 (from 44.5% up to 70.5%), and then it maintained a relatively stable proportion after 8 or more passages when grown at clonal density. Data represent mean ± SD of three independent experiments.
(TIF)

Table S1 **Limiting dilution assay for spheroid colony formation.** Tumorsphere cells and adherent cells were counted by cytometer, original cell solutions with the concentration of 500 cells/ml were inoculated in 48-well ultra-low attachment plates by 100 ul/well i.e. estimated cell number per well was 50 cells. Limiting dilution of cell solutions were carried out by the ratio of 1/5, 1/10 and 1/50 for tumorsphere cells and adherent cells, and the cells were inoculated in 48 wells respectively. After 3 weeks culture, spheroid colonies were counted in each group. The results showed that approximately 27–35% of tumorsphere cells could produce spheroid colonies, while less than 5% of adherent cells could generate spheroid colonies after 3 weeks culture. Therefore, within the tumorsphere cells, we could estimate that the spheroid-forming cell population comprised a maximum of 35%.
(DOC)

Table S2 **Limiting dilution assay for spheroid colony formation.** Mice were only treated cyclopamine at the right rear flank or control tomatidine at the left rear flank (twice a week for 3 weeks) when tumors from HGC-27 tumorsphere cells reached about 2 mm, after 3 weeks, we performed a limiting dilution assay for spheroids formation using tumor cells treated with cyclopamine and treated with tomatidine, respectively. Approximately 9–15% of cells from xenotransplanted tumor treated with tomatidine could produce spheroids, while less than 2.5% of cells from xenotransplanted tumor treated with cyclopamine could generate spheroids. Therefore, blocking SHH pathway in vivo could reduce the numbers of gastric CSLCs. Tom = tomatidine, Cyc = cyclopamine.
(DOC)

Table S3 **Case description and tumor features.** The human fresh, sterile gastric cancer tissue specimens were obtained in accordance with the ethical standards of the institutional committee on human experimentation from 15 patients (age range 70–85 years) undergoing a gastric cancer resection, after obtaining informed consent from the patients.
(DOC)
