# Supplementary figures and images for: Sonic Hedgehog Pathway Is Essential for Maintenance of Cancer Stem-Like Cells in Human Gastric Cancer
Source: PLoS One. 2011 Mar 4;6(3):e17687. doi: 10.1371/journal.pone.0017687 (PMC3048871; doi:10.1371/journal.pone.0017687)

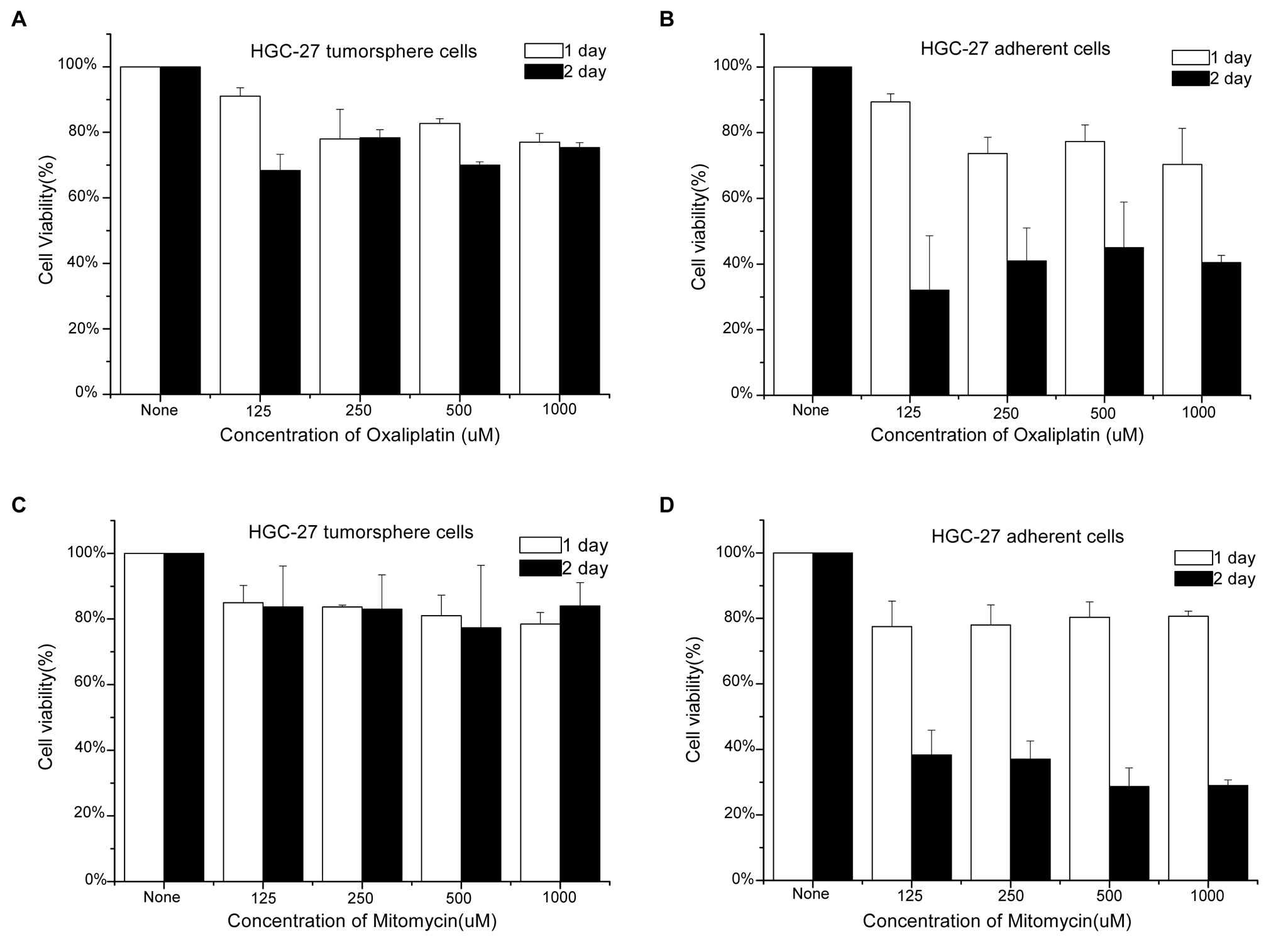

Supplement: Figure S1 — The chemoresistance of HGC-27 tumorsphere cells and adherent cells to different concentrations of drugs. Figure showed that HGC-27 tumorsphere cells demonstrated significantly greater resistance to different concentrations (125µM, 250µM, 500µM and 1000µM) of drugs (Oxaliplatin, Mitomycin) compared with the adherent cells after 48 hours, however, both tumorsphere cells and adherent cells did not show significantly chemoresistance to drugs in a dose-dependent fashion. White bars = 1 day. Black bars = 2 day. (TIF) [file pone.0017687.s001.tif]

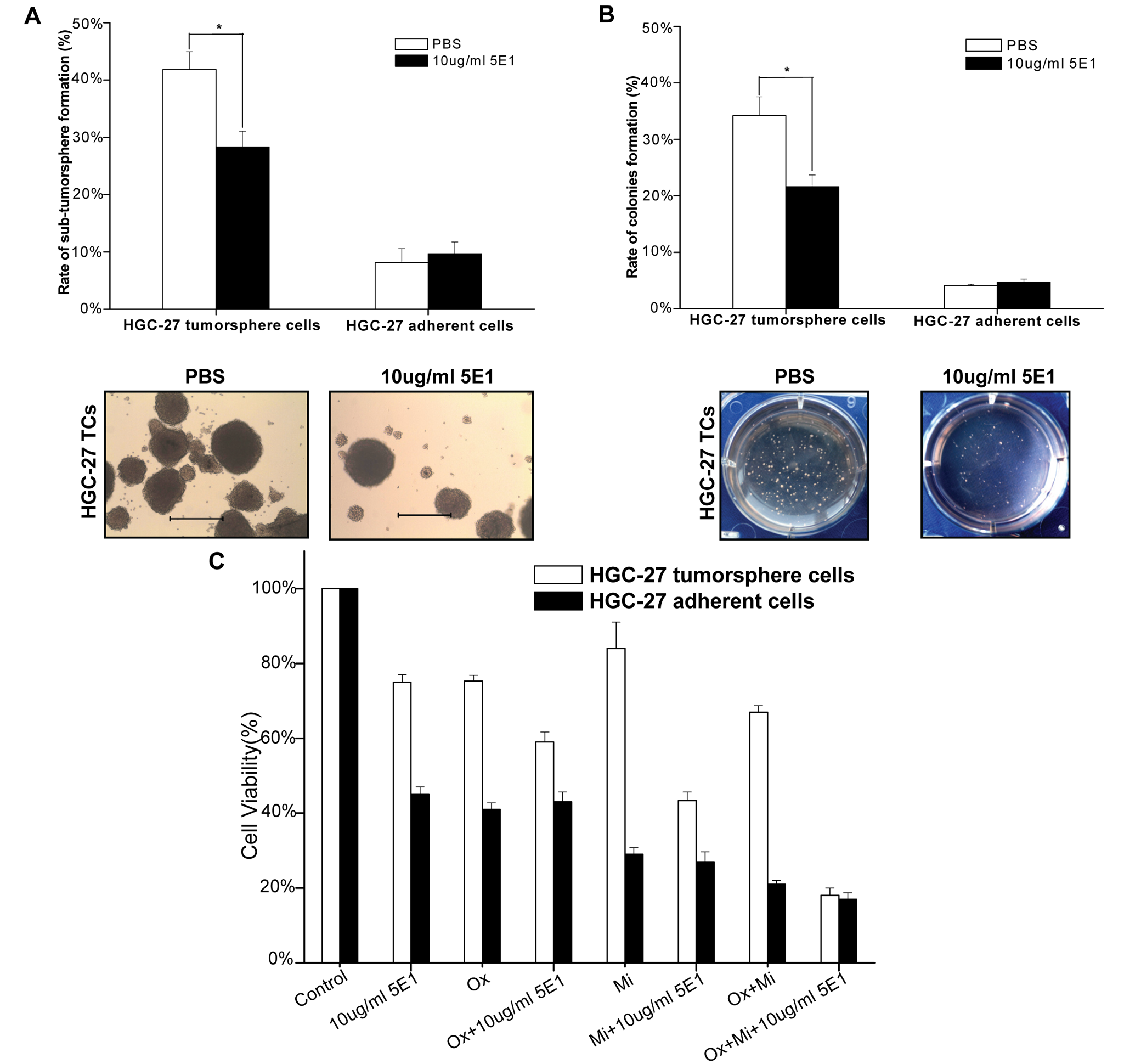

Supplement: Figure S2 — Effect of anti-SHH antibody 5E1 on the self-renewing capacity and chemoresistance. Figure showed that 10µg/ml 5E1 treatment did not led to decrease in the capacity for the formation of sub-tumorspheres in HGC-27 adherent cells but a significantly greater reduction (13.5%) in HGC-27 tumorsphere cells. Similarly, the capacity to form colonies in soft agar was reduced more in the HGC-27 tumorsphere cells (12.6%) relative to the adherent cells. The effect of 5E1 on cell death to HGC-27 adherent cells was more significance than tumorsphere cells, but no such synergistic effect was observed in adherent cells after exposure to drugs with 5E1 for 48 hours. When 5E1 was followed by drugs in HGC-27 tumorsphere cells, the treatment resulted in a significantly enhance overall cell death rate. Best results were obtained when 5E1 was combined with Oxaliplatin plus Mitomycin. Control group is PBS. * = P<0.05. Bars = 1000µm. (TIF) [file pone.0017687.s002.tif]

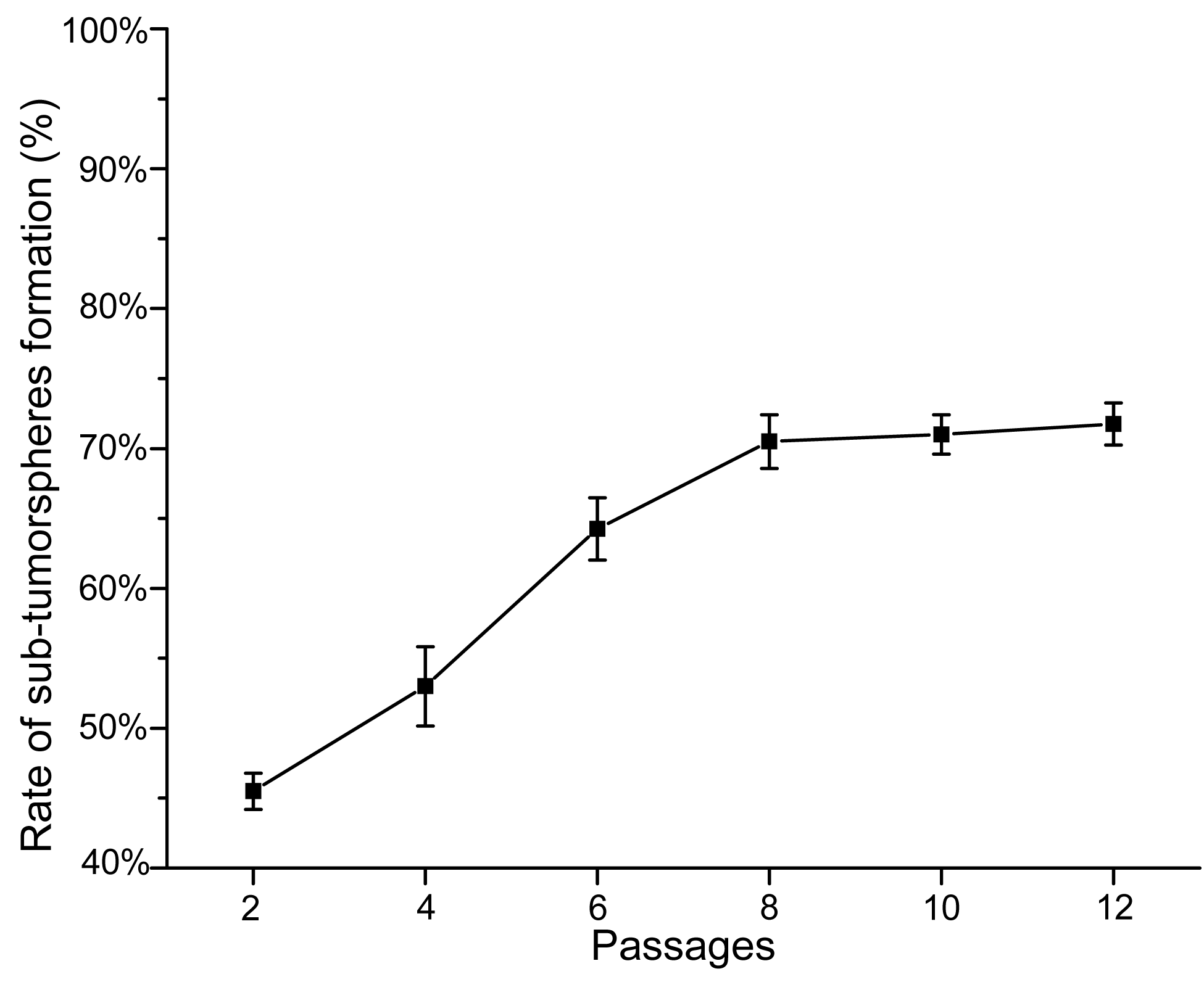

Supplement: Figure S3 — The self-renewal ability to form sub-tumorspheres of HGC-27 tumorsphere cells during serial passages. Figure showed that the self-renewal ability to form sub-tumorspheres of HGC-27 tumorsphere cells had a increase at passages 2 to 8 (from 44.5% up to 70.5%), and then it maintained a relatively stable proportion after 8 or more passages when grown at clonal density. Data represent mean ± SD of three independent experiments. (TIF) [file pone.0017687.s003.tif]
