# Supplementary material for: Sonic Hedgehog Pathway Is Essential for Maintenance of Cancer Stem-Like Cells in Human Gastric Cancer
Source: PLoS One. 2011 Mar 4;6(3):e17687. doi: 10.1371/journal.pone.0017687 (PMC3048871; doi:10.1371/journal.pone.0017687)
Supplement: Table S1 — Limiting dilution assay for spheroid colony formation. Tumorsphere cells and adherent cells were counted by cytometer, original cell solutions with the concentration of 500 cells/ml were inoculated in 48-well ultra-low attachment plates by 100 ul/well i.e. estimated cell number per well was 50 cells. Limiting dilution of cell solutions were carried out by the ratio of 1/5, 1/10 and 1/50 for tumorsphere cells and adherent cells, and the cells were inoculated in 48 wells respectively. After 3 weeks culture, spheroid colonies were counted in each group. The results showed that approximately 27–35% of tumorsphere cells could produce spheroid colonies, while less than 5% of adherent cells could generate spheroid colonies after 3 weeks culture. Therefore, within the tumorsphere cells, we could estimate that the spheroid-forming cell population comprised a maximum of 35%. (DOC) [file pone.0017687.s004.doc]

Table S1: Limiting dilution assay for spheroid colony formation.

|  | HGC-27 tumorsphere cells | | | | HGC-27 adherent cells | | | |
| --- | --- | --- | --- | --- | --- | --- | --- | --- |
| Dilution ratio | 1/1 | 1/5 | 1/10 | 1/50 | 1/1 | 1/5 | 1/10 | 1/50 |
| Cells/well | 50 | 10 | 5 | 1 | 50 | 10 | 5 | 1 |
| Colonies+ wells | 48 | 37 | 27 | 13 | 16 | 10 | 4 | 1 |
| Total colonies | 847 | 157 | 71 | 13 | 125 | 18 | 6 | 1 |
| Total cells | 2400 | 480 | 240 | 48 | 2400 | 480 | 240 | 48 |
| Colonies/total cells(%) | 35.3 | 32.7 | 29.6 | 27.1 | 5.2 | 3.8 | 2.5 | 2.1 |
