# Supplementary material for: Sonic Hedgehog Pathway Is Essential for Maintenance of Cancer Stem-Like Cells in Human Gastric Cancer
Source: PLoS One. 2011 Mar 4;6(3):e17687. doi: 10.1371/journal.pone.0017687 (PMC3048871; doi:10.1371/journal.pone.0017687)
Supplement: Table S2 — Limiting dilution assay for spheroid colony formation. Mice were only treated cyclopamine at the right rear flank or control tomatidine at the left rear flank (twice a week for 3 weeks) when tumors from HGC-27 tumorsphere cells reached about 2 mm, after 3 weeks, we performed a limiting dilution assay for spheroids formation using tumor cells treated with cyclopamine and treated with tomatidine, respectively. Approximately 9–15% of cells from xenotransplanted tumor treated with tomatidine could produce spheroids, while less than 2.5% of cells from xenotransplanted tumor treated with cyclopamine could generate spheroids. Therefore, blocking SHH pathway in vivo could reduce the numbers of gastric CSLCs. Tom = tomatidine, Cyc = cyclopamine. (DOC) [file pone.0017687.s005.doc]

Table S2: Limiting dilution assay for spheroid colony formation.

|  | Cells from xenotransplanted tumor treated with tom | | | | Cells from xenotransplanted tumor treated with cyc | | | |
| --- | --- | --- | --- | --- | --- | --- | --- | --- |
| Dilution ratio | 1/1 | 1/5 | 1/10 | 1/50 | 1/1 | 1/5 | 1/10 | 1/50 |
| Cells/well | 50 | 10 | 5 | 1 | 50 | 10 | 5 | 1 |
| Colonies+ wells | 48 | 32 | 25 | 4 | 30 | 6 | 1 | 0 |
| Total colonies | 365 | 66 | 25 | 4 | 60 | 6 | 1 | 0 |
| Total cells | 2400 | 480 | 240 | 48 | 2400 | 480 | 240 | 0 |
| Colonies/total cells(%) | 15.2 | 13.8 | 10.5 | 9.1 | 2.5 | 1.3 | 0.4 | 0 |
