# Supplementary material for: Sonic Hedgehog Pathway Is Essential for Maintenance of Cancer Stem-Like Cells in Human Gastric Cancer
Source: PLoS One. 2011 Mar 4;6(3):e17687. doi: 10.1371/journal.pone.0017687 (PMC3048871; doi:10.1371/journal.pone.0017687)
Supplement: Table S3 — Case description and tumor features. The human fresh, sterile gastric cancer tissue specimens were obtained in accordance with the ethical standards of the institutional committee on human experimentation from 15 patients (age range 70–85 years) undergoing a gastric cancer resection, after obtaining informed consent from the patients. (DOC) [file pone.0017687.s006.doc]

Table S3. Case description and tumor features.

| Patient | Age/Sex | Site | Stage | Spheres formation |
| --- | --- | --- | --- | --- |
| P1 | 73/M | antrum | ⅡB | yes |
| P2 | 80/M | corpus | ⅡB | yes |
| P3 | 75/F | antrum | ⅢA | yes |
| P4 | 78/F | corpus | ⅢB | yes |
| P5 | 76/F | fundus | ⅢC | yes |
| P6 | 78/M | pylorus | ⅢC | yes |
| P7 | 85/F | fundus | ⅡB | no |
| P8 | 77/F | corpus | ⅡB | yes |
| P9 | 83/F | antrum | ⅢB | yes |
| P10 | 76/M | pylorus | ⅡB | no |
| P11 | 76/M | antrum | ⅢB | yes |
| P12 | 70/F | antrum | ⅢC | yes |
| P13 | 78/M | fundus | ⅡB | yes |
| P14 | 77/M | fundus | ⅢB | yes |
| P15 | 76/F | corpus | ⅡB | yes |
